# Unveiling microbial biomarkers of ruminant methane emission through machine learning

**DOI:** 10.3389/fmicb.2023.1308363

**Published:** 2023-12-08

**Authors:** Chengyao Peng, Ali May, Thomas Abeel

**Affiliations:** ^1^Delft Bioinformatics Lab, Delft University of Technology, Delft, Netherlands; ^2^dsm-firmenich, Science & Research, Delft, Netherlands; ^3^Infectious Disease and Microbiome Program, Broad Institute of MIT and Harvard, Cambridge, MA, United States

**Keywords:** rumen microbiome, enteric methane, ruminants, machine learning, regression, feature selection, precision animal feed

## Abstract

**Background:**

Enteric methane from cow burps, which results from microbial fermentation of high-fiber feed in the rumen, is a significant contributor to greenhouse gas emissions. A promising strategy to address this problem is microbiome-based precision feed, which involves identifying key microorganisms for methane production. While machine learning algorithms have shown success in associating human gut microbiome with various human diseases, there have been limited efforts to employ these algorithms to establish microbial biomarkers for methane emissions in ruminants.

**Methods:**

In this study, we aim to identify potential methane biomarkers for methane emission from ruminants by employing regression algorithms commonly used in human microbiome studies, coupled with different feature selection methods. To achieve this, we analyzed the microbiome compositions and identified possible confounding metadata variables in two large public datasets of Holstein cows. Using both the microbiome features and identified metadata variables, we trained different regressors to predict methane emission. With the optimized models, permutation tests were used to determine feature importance to find informative microbial features.

**Results:**

Among the regression algorithms tested, random forest regression outperformed others and allowed the identification of several crucial microbial taxa for methane emission as members of the native rumen microbiome, including the genera *Piromyces, Succinivibrionaceae UCG-002*, and *Acetobacter*. Additionally, our results revealed that certain herd locations and feed composition markers, such as the lipid intake and neutral-detergent fiber intake, are also predictive features for methane emissions.

**Conclusion:**

We demonstrated that machine learning, particularly regression algorithms, can effectively predict cow methane emissions and identify relevant rumen microorganisms. Our findings offer valuable insights for the development of microbiome-based precision feed strategies aiming at reducing methane emissions.

## 1 Introduction

Cattle production emits an excessive amount of greenhouse gas (GHG). Strikingly, cow enteric methane emissions alone can account for 3.3% of total anthropogenic greenhouse gas (GHG) emissions and therefore are significant contributors to global warming (Knapp et al., 2014). The main GHG in cow enteric emissions is methane (CH_4_), which is produced by microbial fermentation of high-fiber feed in the rumen, the digestive center in ruminants. The rumen microbiome is a complex community comprising thousands of different microorganisms, including bacteria, archaea, fungi, and protozoa. These microorganisms play a crucial role in breaking down indigestible polysaccharides into volatile fatty acids (e.g., acetate, butyrate, and propionate), which serve as essential energy source for their ruminant host animals. This microbial fermentation process also generates by-products such as carbon dioxide (CO_2_), hydrogen (H_2_), and methyl compounds (Jouany, 1991), which can be utilized by methanogens, a group of anaerobic archaea in the rumen microbiome, to produce methane. The dominant methanogen genera in ruminants include *Methanobrevibacter, Methanosphaera*, and *Methanomassiliicoccus* (Jeyanathan et al., 2011).

**Figure 1:**
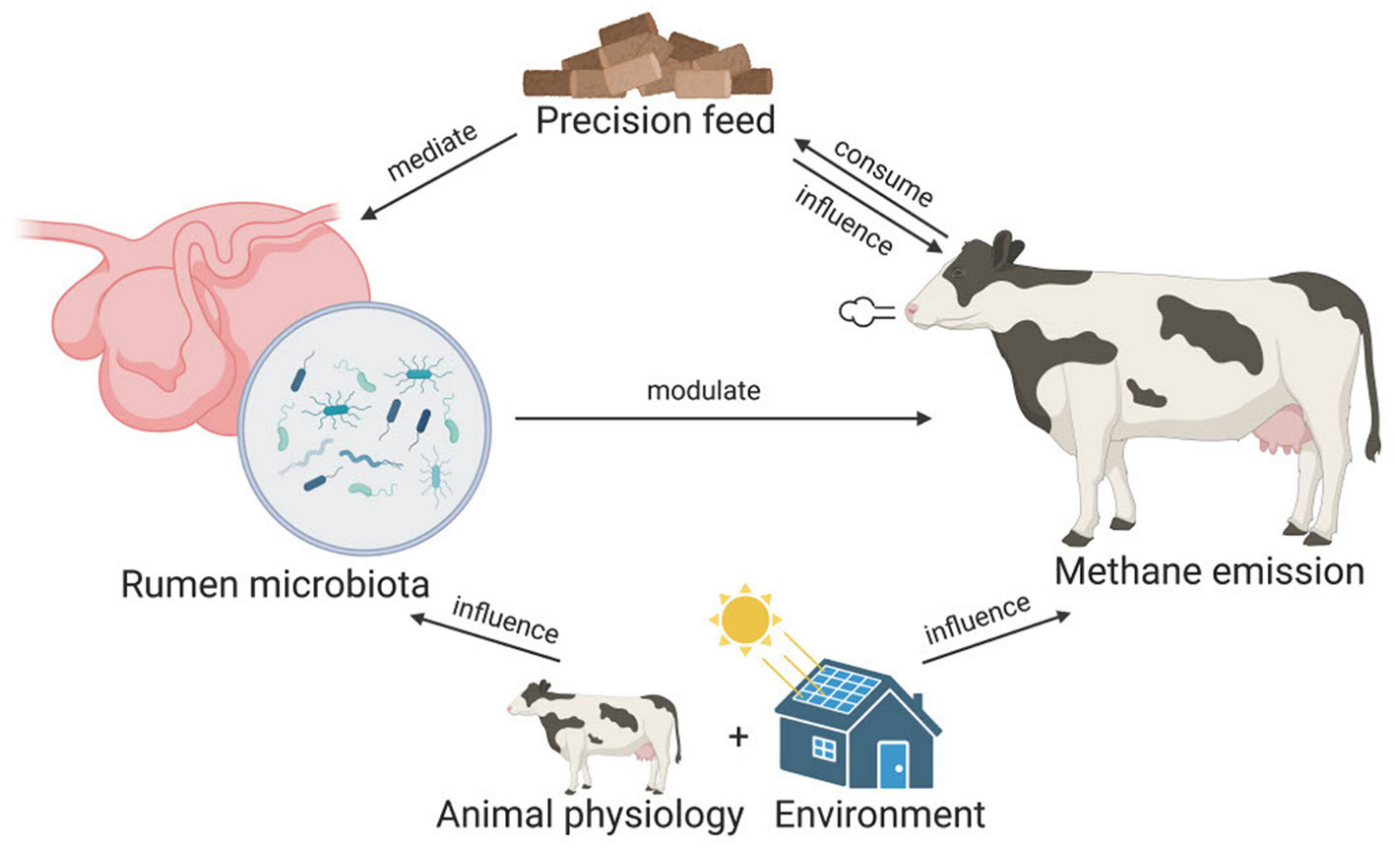
The interconnected relationships of feed, rumen microbiome, animal physiology, herd location, and methane emission. In microbiome-based precision feed, ruminants consume optimized feed that mediates the rumen microbiome compositions, which in turn modulate the enteric methane emission. Besides mediating the rumen microbiome, the feed can also directly influence methane emissions. Animal physiology such as the lactation stage of the animal and the living environment can also influence both the rumen microbiome and the methane emission of the ruminants.

To reduce methane emission in ruminants, microbiome-based precision feed has been proposed as a promising strategy (Huws et al., 2018; Goopy, 2019; Smith et al., 2022). Microbiome-based precision feed involves optimizing animal feed to modulate rumen microbiome compositions and functions to inhibit methanogenesis. However, the development of microbiome-based precision feed relies on our understanding of the rumen microbiome. Specifically, the microorganisms that are involved in methanogenesis need to be identified. To achieve this, aside from archaea, which can be direct methane producers, different studies have associated the abundance of bacteria, fungi, and protozoa with methane emissions. These studies typically use statistical tests (e.g., *t*-tests) and linear methods (e.g., partial least squared regression and linear mixed model) to identify microbes that are significantly different in abundance between cows with distinct emission profiles, e.g., high vs. low emitters (Wallace et al., 2015; Kamke et al., 2016; Difford et al., 2018; Ramayo-Caldas et al., 2020). However, such methods may not be adequate to capture the nonlinearity and complexity in the microbiome data (Quinn et al., 2021). In addition, because of the high dimensionality in microbiome data, the reported list of associated taxa in previous studies can be unworkably long. For example, using statistical analysis, a recent study reported 395 taxa that were significantly correlated with methane emissions (Savin et al., 2022). Last but not least, some studies fail to account for biological variables such as animal physiology and the living environment when examining the relationship between the rumen microbiome and methane emission. These variables, which are interconnected with rumen microbiome and methane emission, should be included in the model. A conceptual visualization of the interconnected relationships of feed, rumen microbiota, animal physiology, herd location and methane emission can be found in Figure 1.

Recently, an increasing number of human microbiome studies have successfully utilized machine learning (ML) algorithms to select relevant microbial features to predict human diseases (Marcos-Zambrano et al., 2021). ML algorithms have several important advantages over traditional statistical methods: (i) ML-based feature selection methods can reduce the dimensionality in microbiome data to prevent overfitting compared to traditional statistical tests and linear models, (ii) ML models such as support vector machines (SVM), k-nearest neighbors (KNN), and random forest (RF) can handle nonlinear relationships in the data, (iii) evaluating model performance is relatively straightforward because ML models are predictive models, and (iv) the prioritization of important microbial features is straightforward with the optimized models. Despite these advantages, no previous studies have systematically investigated ML algorithms to facilitate our understanding of methane emissions in cows.

In this paper, we aim to address this gap by benchmarking commonly-used feature selection methods and regression algorithms, which have proven useful in human microbiome studies, to identify microbial biomarkers for methane emissions of Holstein cows. To this end, we taxonomically analyzed two large rumen microbiome datasets, corrected for existing batch effects and identified biological host metadata variables that can influence methane emission, including the herd location, lactation stage, and the individual intake of different feed compounds. Subsequently, we benchmarked four common feature selection methods and six regressors to predict methane emission based on the rumen microbiome compositions and the host biological metadata. The hyperparameters of these regressors and the feature selection methods were optimized through a bootstrap sampling strategy and 10-fold cross-validation with 100 repetitions. Afterward, we investigated the important features in the optimized model to identify the important microbial features for methane emission. Our study provides a systematical evaluation of different ML regression algorithms for methane emission prediction in Holstein cows. Our findings in the feature importance test will contribute to the development of microbiome-based precision feed to reduce the environmental impact of the livestock industry.

## 2 Materials and methods

### 2.1 Datasets

Two large rumen amplicon sequencing datasets from Difford et al. (2018) and Wallace et al. (2019) with methane emission (g/d) metadata were retrieved under ENA Project Accession ERP110230 and PRJNA517480, respectively. In the rest of the manuscript, these two datasets are referred to as the “Difford dataset” and the “Wallace dataset,” respectively. The amplicon sequencing samples with missing metadata were excluded in our study, resulting in a total of 713 Holstein cows from the Difford dataset and 816 cows from the Wallace dataset. The Difford dataset only measured archaea and bacteria for the rumen microbiome, while the Wallace dataset profiled archaea, bacteria, fungi, and protozoa. For technical metadata variables, the Difford dataset documented the sequencing instrument and sequencing batch for each microbiome sample. The Wallace dataset recorded the feed intake methods and methane emission measurement methods. As for biological metadata variables, we only considered those potential confounding factors based on literature, which should also have no missing data in the datasets, i.e., herd location and lactation stage for cows. Additionally, the Wallace dataset recorded the intake information of several feed compositions for each animal, including dry matter (DM), organic matter (OM), crude protein (CP), NDF (neutral-detergent fiber), lipid, acid insoluble ash (AIA), and starch.

### 2.2 Methods

**Figure 2:**
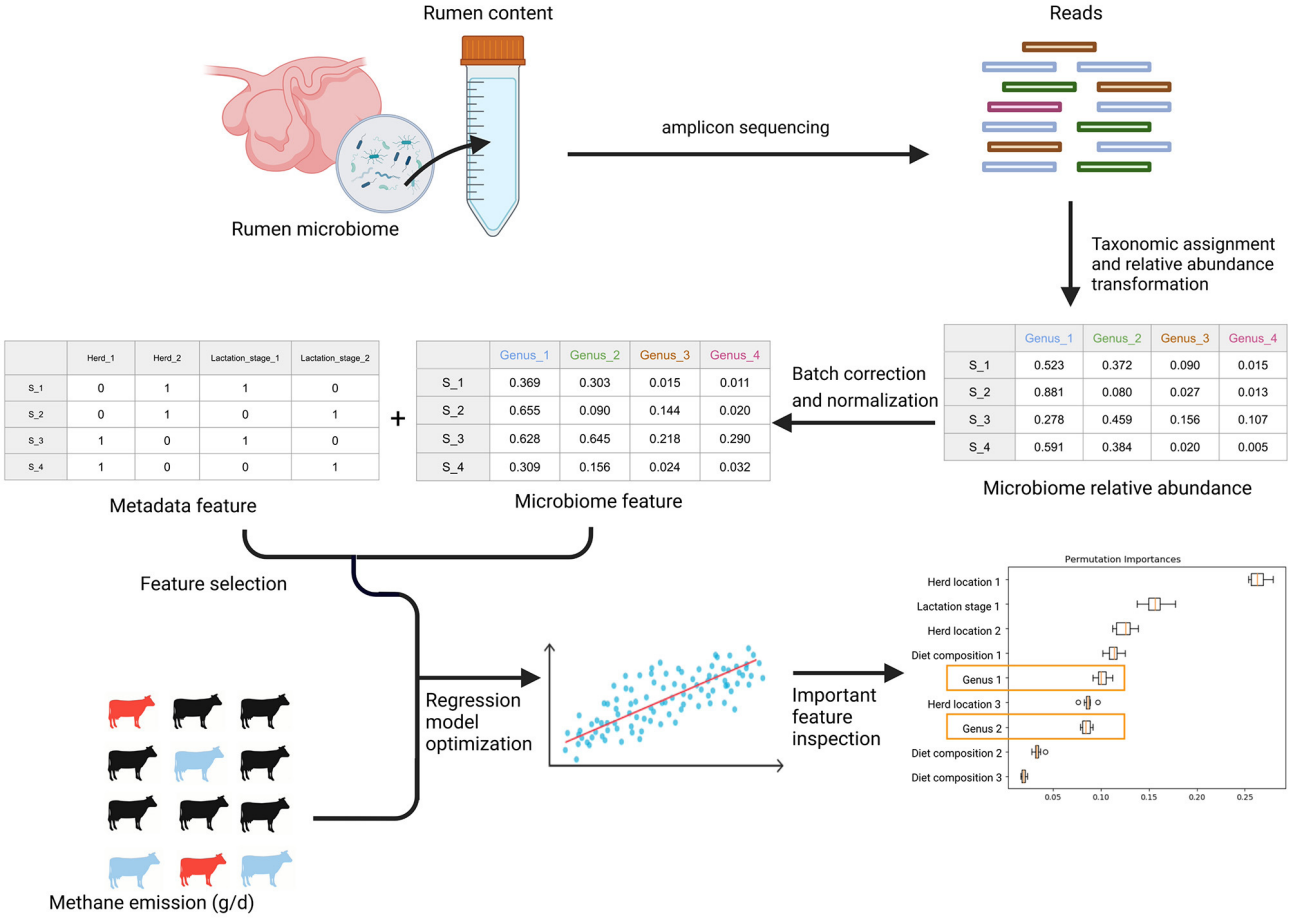
The general workflow. After amplicon sequencing of rumen microbiome samples from rumen content extracts, the workflow begins with taxonomic composition analysis. After the taxonomic assignment and relative abundance transformation, batch correction is applied to remove technical variations in the microbiome data. Later, the microbiome features were obtained after normalization of each batch corrected OTU. The confounding biological metadata variables were identified by statistical tests. With microbiome and metadata features alone and in combination, we optimized six regression models and the corresponding feature selection method to predict methane emission from individual cows through a stratified bootstrap sampling strategy and 10-fold cross-validation with 100 repetitions. In the end, we calculated permutation feature importance to understand how the optimized algorithms leverage different features to predict methane emission and identify the important microbial features.

An overall workflow from the taxonomic composition analysis, batch correction, feature table generation, feature selection, and regressor optimization and permutation feature importance can be found in Figure 2. The details of each step are described below.

#### 2.2.1 Taxonomic analysis of the rumen microbiome

For both the Difford dataset and Wallace dataset, FastQC v0.11.7 (Andrews et al., 2010) and Trimmomatic v0.39 (Bolger et al., 2014) were used to assess the data quality and to trim low-quality bases. Next, Kraken2 v2.1.2 was used to profile the taxonomic compositions in all microbiome samples at genus-level OTUs (Wood et al., 2019) using the SILVA release 138.1 (Quast et al., 2012). The less frequent OTUs that showed up in <50% of the animals in each dataset were filtered out. The raw counts of OTUs were transformed into relative abundance.

To investigate the potential of combining the samples from both datasets, we additionally created a merged dataset that consists of shared genus-level OTUs by the two original datasets, referred to as the “Merged dataset.” Likewise, less frequent OTUs that showed up in <50% of the animals were filtered out and relative abundance was calculated.

#### 2.2.2 Identification and correction of batch effects in rumen microbiome

To identify the possible bias introduced by technical factors, we performed a dimension reduction using UMAP from the Python library umap-learn v0.5.3 (McInnes et al., 2018) to visualize how technical factors correlate with microbiome batch effects. As a result, we used Combat from Python library pyComBat v0.4.4 (Johnson et al., 2007) to correct for the existing batch effects and visualized the corrected outcomes.

Since some of the used regressors in our downstream analysis assume a Gaussian distribution of the data, we normalized the batch-corrected OTUs in all datasets using z-score normalization.

#### 2.2.3 Transforming confounding biological metadata variables

Existing studies have shown that herd location, lactation stage, and feed intake composition can influence methane emission from cows (Gibbs et al., 1989; Cottle et al., 2011; Lyons et al., 2018). To confirm their possible confounding effects in our data, we tested their correlation with methane emission. In particular, we used the Kruskal–Wallis test to test if there is a significant difference in methane emission between different groups of herd location and lactation stage. For the numerical variables of feed composition intake in the dataset Wallace, we performed a pairwise Spearman correlation and a following two-tailed *t*-test. All *p*-values were adjusted using Bonferroni correction to control the type I errors for multiple testing. The categorical host variables that were tested to be significant were one-hot encoded and the numerical variables were normalized by z-score normalization for further analysis.

#### 2.2.4 Feature table and target generation

To investigate the predictive power of microbiome and biological metadata for methane emission (i) separately, and (ii) jointly; we created three feature tables for each dataset: one with only microbiome compositions, one with only metadata variables, and another that included both microbiome features and metadata variables. The tables were named according to the dataset and feature they contained, i.e., “Difford: microbiome,” “Difford: metadata,” “Difford: microbiome+metadata,” “Wallace: microbiome,” “Wallace: metadata,” “Wallace: microbiome+metadata,” “Merged: microbiome,” “Merged: metadata,” and “Merged: microbiome+metadata.”

#### 2.2.5 Optimizing feature selection methods and prediction models

To predict methane emission based on the feature tables generated across all datasets, we built a machine learning pipeline that included four common feature selection methods and six regression models, using the Python library scikit-learn v1.3.0 (Pedregosa et al., 2011). The included feature selection methods were f-statistics, lasso regression feature importance, mutual information and random forest feature importance. The six regression models include linear regression, linear support vector regressor (linear SVR), elastic net regression, kernel support vector regressor (kernel SVR), K-Nearest Neighbor regression (KNN regression), and random forest regression (RF regression).

To find the best strategy to predict methane emission, the samples in all tables were split into training and test sets (80:20) by a bootstrap sampling strategy with 100 repetitions. The ranges or values for different hyperparameters in each regression algorithm were listed in Supplementary Table 1. To find the optimal hyperparameters for each regression model in each training set, we used Bayesian optimization provided by Optuna (Akiba et al., 2019) with a 10-fold cross-validation strategy. The feature selection method optimization was nested into the cross-validation procedure. The evaluation metric was *r*^2^ score. With the optimized feature selection method and hyperparameters for each regression model, we assess the model performance by the according test sets.

#### 2.2.6 Permutation feature importance calculation

To understand how the optimized algorithms leverage both microbiome and metadata features to predict methane emission during the test, we performed feature permutation importance tests for each feature with 100 repetitions. The resulting model performance decrease was measured to assign an importance score to each feature.

The microbiome features for which the permutation led to an *r*^2^ decrease >0.01 were considered important microbial biomarkers for methane production. We further visualized the relationship between the transformed abundance of these important genera and methane emission in the corresponding dataset. If the relationship appears to be monotonic, to gain quantitative insight, a pairwise Spearman correlation coefficient was calculated, followed by a two-tail *t*-test.

## 3 Results

### 3.1 Taxonomic profiling of 1,529 rumen microbiomes

To assess the metagenomic diversity across the two data collections in our study, we taxonomically profiled the samples with complete metadata in both Difford (*n* = 713) and Wallace (*n* = 816) datasets on the genus level. A merged dataset was created from the shared OTUs from the two original datasets. The low-occurrence OTUs in each dataset were filtered out prior to further analysis. In all datasets, these removed OTUs accounted for <0.5% of reads on average across the samples. As a result, in the Difford dataset, we acquired 429 genus-level OTUs (415 bacterial and 14 archaeal), while in the Wallace dataset, we obtained 163 genus-level OTUs (150 bacterial, 7 archaeal, 11 protozoan, and 5 fungal). The Merged dataset comprised 182 genus-level OTUs (176 bacterial and 6 archaeal).

**Figure 3:**
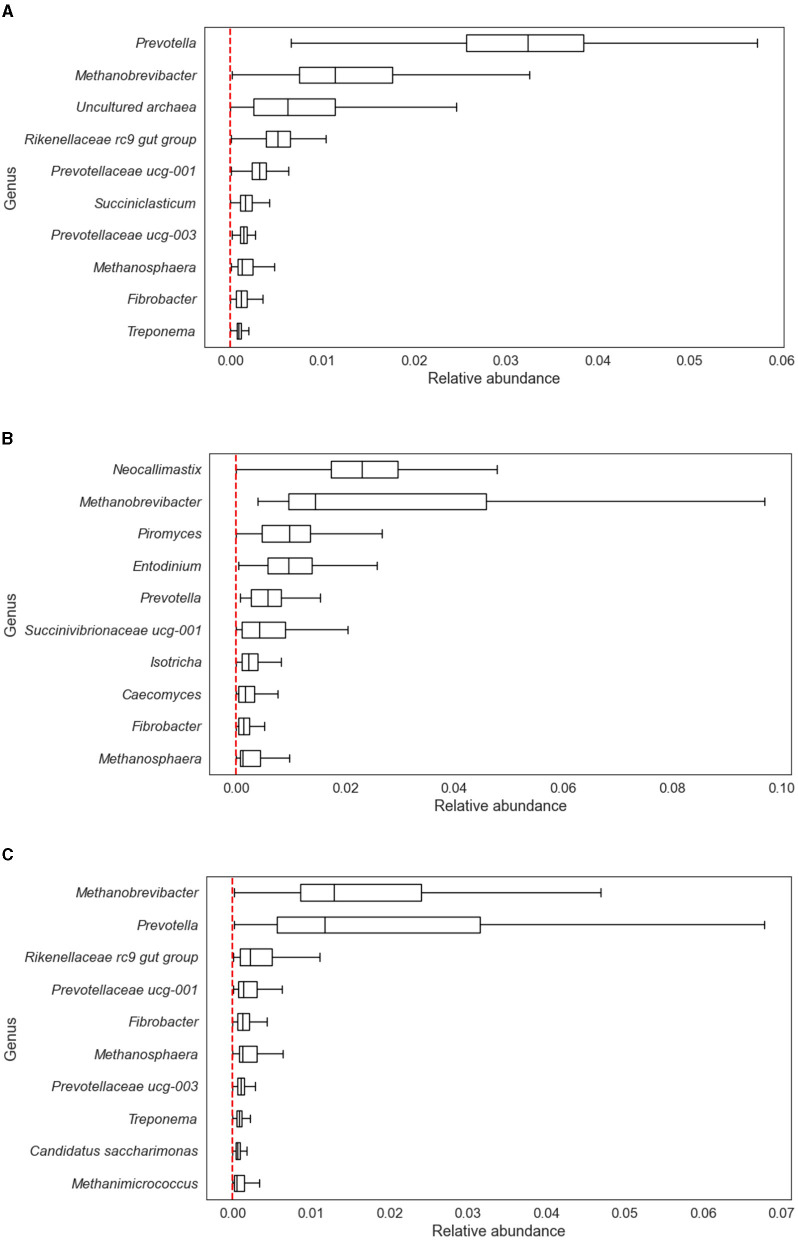
Relative abundance of ten most abundant genus-level OTUs. **(A)** In the Difford dataset. **(B)** In the Wallace dataset. **(C)** In the Merged dataset. The x-axis shows the relative abundance and the y-axis displays the genus names. The genus-level OTUs were ranked by the median relative abundance across all samples in each dataset.

Figure 3 provides an overview of the ten most abundant OTUs in each dataset, ranked by the median of their relative abundance across all samples. As shown, notably, *Methanobrevibacter* and *Methanosphaera*, two hydrogenotrophic methanogens, were among the ten most abundant OTUs in all three datasets. In the Merged dataset, *Methanimicrococcus*, another hydrogenotrophic methanogen, was the tenth most abundant OTU.

### 3.2 Batch effects in the profiled rumen microbiome data were addressed

After the normalization of individual OTUs gained in the taxonomic profiling, we visualized potential batch effects in each dataset using dimensionality reduction by UMAP. As shown in Supplementary Figure 1A, we observed that the sequencing batches and sequencing machines introduced obvious batch effects in the Difford dataset. After removing the batch effects on the level of the sequencing batch, we were able to remove the variation between all the batches (Supplementary Figure 1B). Similarly, we also identified batch effects in the Wallace dataset, as demonstrated in Supplementary Figure 1C. To understand what factors introduced the batch effects, we examined all the recorded metadata variables but unfortunately, none correlated with the apparent pattern. Accordingly, we corrected the batch effects for the two distinctly separable clusters, named “Unknown cluster 1” and “Unknown cluster 2” and the batch effects were alleviated (Supplementary Figure 1D). In the Merged dataset, as anticipated, we observed similar batch effect patterns as in the two original datasets, as plotted in Supplementary Figure 1E. After batch correction, the dissimilarity among different batches from the two datasets decreases noticeably (Supplementary Figure 1F).

### 3.3 Metadata variables are significantly associated with methane emission

To confirm that we should include the recorded biological metadata factors that could have confounding effects into regression, we investigated their relationships with methane emission in our datasets.

Initially, we investigated the impact of herd location and lactation stage in methane emission levels. Using a Kruskal–Wallis test, we identified a significant association between the herd location and methane emission (*p* < 0.001) in all three datasets: Difford, Wallace, and Merged (Table 1). For the lactation stage, such association was significant in the Difford and Wallace dataset. These findings indicate that these two variables indeed could have confounding effects. Therefore, in further analysis, we one-hot encoded the herd location as metadata features in all datasets and the lactation stage in the Difford and Merged dataset.

**Table 1:** Kruskal–Wallis test for associating herd location and lactation stage with methane emission (g/d) across all datasets.

**Datasets**	**Statistics**	**Herd location**	**Lactation stage**
Difford dataset	Chi^2^	30.86	10.59
	df	5	2
	*p*	< 0.001	0.012
Wallace dataset	Chi^2^	349.85	0.71
	df	4	2
	*p*	< 0.001	0.702
Merged dataset	Chi^2^	531.05	11.16
	df	10	2
	*p*	< 0.001	0.012

The *p*-values were adjusted with Bonferroni correction.

Aside from the Kruskal–Wallis test, we also visualized the methane emission distribution across the two categorical variables: herd locations and lactation stages. Notably, as shown in Supplementary Figure 2, animals from specific herd locations, such as “Herd 5” in the Difford dataset and “Herd IT1” in the Wallace dataset, stood out by having either higher or lower methane emitters. Given the seemingly large impact of herd location on methane emission, we stratified the later train-test split in the ML pipeline to generate representative test sets.

To identify significant correlations between the intake of feed components recorded exclusively in the Wallace dataset with animal methane emission, we calculated the pairwise Spearman correlation coefficients and conducted the corresponding two-tailed *t*-test. The resulting coefficients and adjusted *p*-values in Table 2 indicate a positive correlation between the feed intake (kg/d) of dry matter (DM), organic matter (OM), crude protein (CP), and neutral-detergent fiber (NDF) with methane emission (g/d). Conversely, the intake of lipid (kg/d) was negatively associated with animal methane emission (g/d).

**Table 2:** Spearman's correlation coefficients between feed composition intake (kg/d) with methane emission (g/d) in the Wallace dataset.

**Datasets**	**Statistics**	**DM**	**OM**	**CP**	**NDF**	**Lipid**	**AIA**	**Starch**
Wallace dataset	Coefficient	0.25	0.25	0.31	0.3	−0.12	0.02	−0.04
	*p*	< 0.001	< 0.001	< 0.001	< 0.001	< 0.001	0.635	0.496

The *p*-values were adjusted with Bonferroni correction.

Based on these results, in further analysis of the Wallace dataset, we included the normalized intake values of these feed components as metadata features in regression.

### 3.4 RF regression performed best in unseen test data

To investigate the efficacy of rumen microbiome and metadata, both alone and in combination, to predict methane emission, we optimized the feature selection method and parameters of all regressors using only microbiome features, metadata features, or a combination of both. The feature table used for each dataset under each condition and the corresponding methane emission table for feature selection and regression can be found in the Supplementary Tables 2, 3. The generated feature tables and the corresponding methane emission table can be found in the Supplementary Tables 2, 3. Model optimization was performed through a bootstrap sampling strategy and 10-fold cross-validation with 100 repetitions. After optimization, we tested the model performance on the corresponding test sets. For each regression model, the average achieved test performance and standard deviation was plotted in Supplementary Figure 3.

Based on these results, we observed that when microbiome features were used, whether alone or together with other biological metadata variables, non-linear regressors such as KNN regression and RF regression consistently outperformed linear regressors. On the other hand, when relying solely on metadata variables, the performance from linear models and non-linear models was similar. In general, RF regression demonstrated superior average performance in all datasets, except for the “Merged: microbiome” dataset, where KNN regression showed a marginal advantage. Specifically, when using both rumen microbiome and metadata variables, RF regression achieved an average *r*^2^ of 0.26, 0.56, 0.42 for the Difford, Wallace, and Merged datasets, respectively in the unseen test sets. As a result, we selected RF regression as the final regression model for further analysis.

### 3.5 Merging the datasets improve the predictive power from microbiome

**Figure 4:**
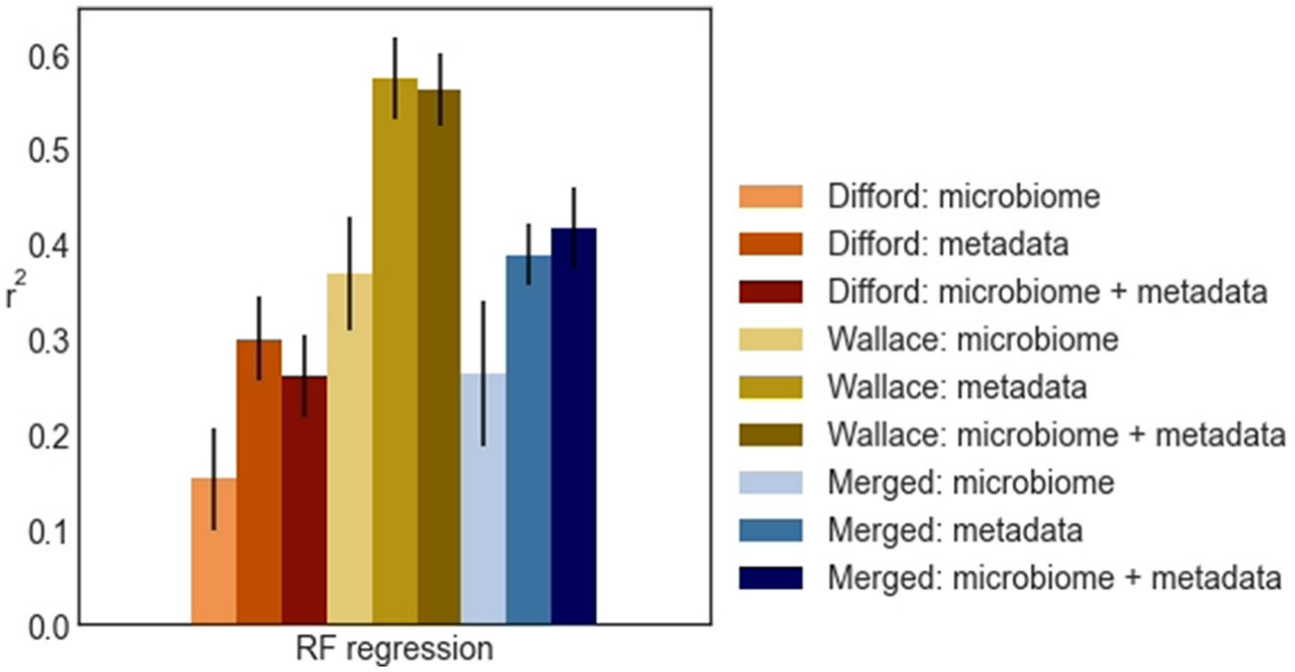
The *r*^2^ random forest regression (RF regression) achieved across Difford, Wallace, and Merged datasets in test. Within each dataset, the random forest regression was optimized with features under either of three conditions: “microbiome” only, “metadata” only, or “microbiome + metadata” jointly. The error bars represent the standard deviations of the achieved test performance across 100 repetitions in the outer loop.

From the test performance of RF regression (Figure 4), we observed that in the Difford and Wallace datasets, the use of metadata features led to slightly better performance compared to using both metadata and microbiome features. In essence, when these two datasets were analyzed in isolation, the addition of microbiome features on top of metadata features did not improve the methane emission prediction.

However, in the Merged dataset, when using microbial features and metadata variables separately, RF regression achieved average *r*^2^ scores of 0.26 and 0.39, respectively. When using these features jointly, RF regression achieved 0.42, a higher average *r*^2^, in the unseen test sets. This indicates that merging the datasets was an essential step for RF regression to effectively learn from the microbiome.

### 3.6 Microbial biomarkers for methane emission: *Piromyces, Succinivibrionaceae UCG-002*, and *Acetobacter*

**Figure 5:**
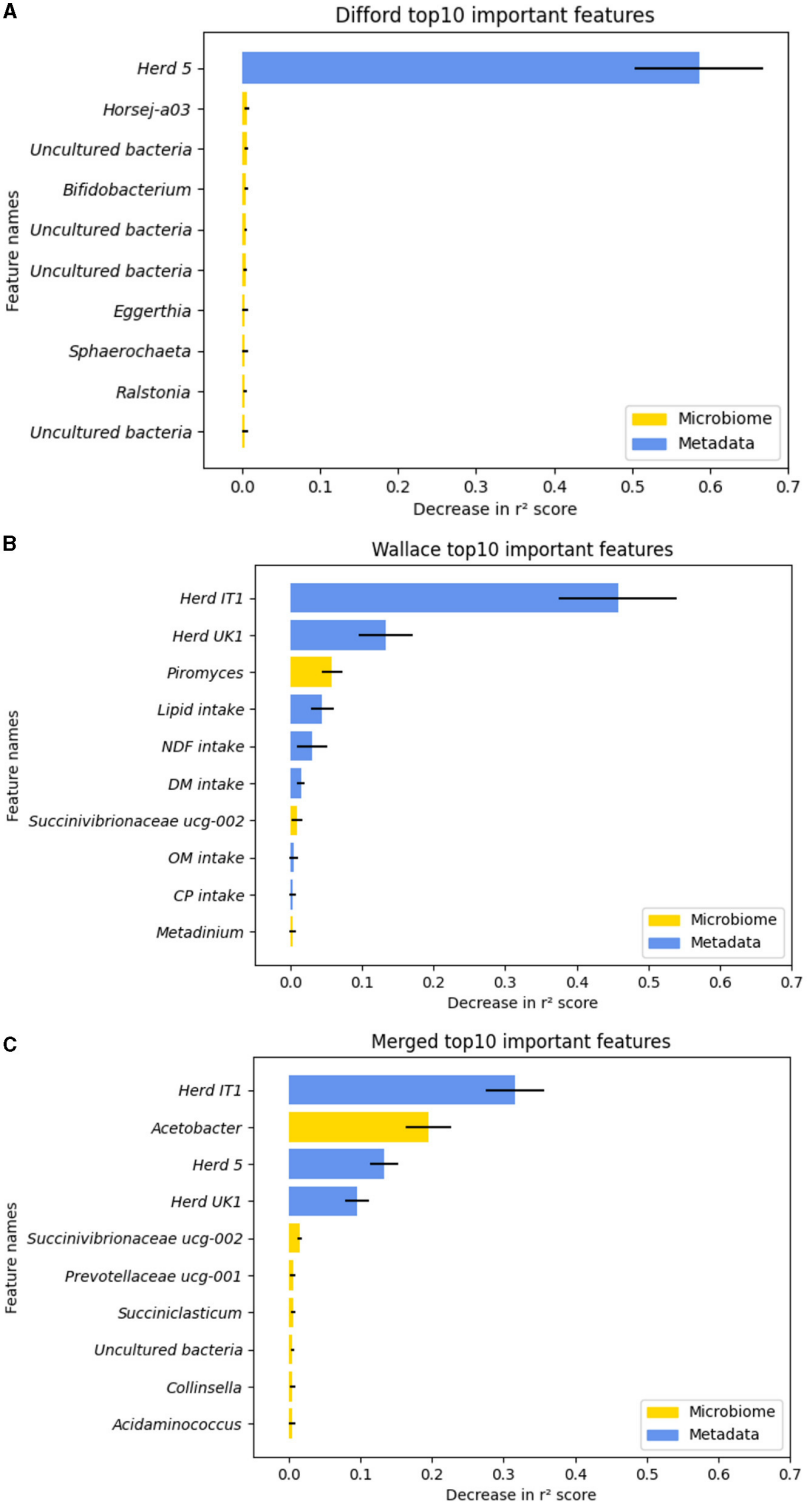
The top-10 important features during the test based on the decrease in *r*^2^ score in permutation tests. **(A)** In the Difford dataset. **(B)** In the Wallace dataset. **(C)** In the Merged dataset. The error bars represent the standard deviations of the decrease in *r*^2^ scores across 100 repetitions of permutation tests of each feature. The microbiome features are indicated by the color yellow, while metadata features are represented by blue.

To understand how RF regression leverage both microbiome and metadata features to predict methane emission, we examined feature permutation importance, using the RF regression model that achieved the highest test performance in “Difford: microbiome+metadata,” “Wallace: microbiome+metadata,” and “Merged:microbiome+metadata.” The permutation for each feature was repeated 100 times to gain a comprehensive evaluation. The top 10 important features in each dataset, which were ranked based on the mean importance score, were plotted in Figure 5.

Regarding the Difford dataset, in the examined RF regression model, 95 features were chosen by lasso-based feature selection, the optimized feature selection method. However, as plotted in Figure 5A, except for the “Herd 5,” the permutation of all the other features led to a decrease in *r*^2^ of < 0.01. In the Wallace dataset, based on the mutual information score, the optimized selection method, 10 features were used for regression. The permutation test identified seven features that resulted in an *r*^2^ decrease greater than 0.01: Herd IT1, Herd IT 2, *Piromyces*, lipid intake, NDF intake, DM intake, *Succinivibrionaceae UCG-002* (Figure 5B). Similarly, for the Merged dataset, the permutation test identified five important features in the optimized model: Herd IT1, *Acetobacter*, Herd 5, Herd UK1 and *Succinivibrionaceae UCG-002* (Figure 5C).

To further understand why the identified microbial features were considered important, we plotted the relationship between their relative abundance with methane emission in their respective datasets (Supplementary Figure 4). As presented, *Piromyces* appears to have a positive association with methane emission. The resulting Spearman correlation coefficient is 0.28 (*p* < 0.001). The transformed abundance of *Succinivibrionaceae UCG-002* and *Acetobacter* seem to form non-monotonic non-linear relationships.

## 4 Discussion

Our analysis revealed several microbial biomarkers for methane emission from Holstein cows, including *Piromyces, Succinivibrionaceae UCG-002*, and *Acetobacter*.

*Piromyces* is estimated to be the most abundant genus of anaerobic fungi in the rumen microbiome (Paul et al., 2018). Experiments have shown that *Piromyces* can effectively degrade glucose and a wide range of plant biomass, including cellulose, crude C3, and C4 bio-energy crops (Solomon et al., 2016). The produced metabolic products, such as H_2_, CO_2_, and formate, can be used by methanogens to produce methane (Sirohi et al., 2010). Natural co-cultures of *Piromyces* and *Methanobrevibacter*, a common genus of methanogenic archaea, have been found in different ruminants, including Holstein cows (Jin et al., 2011; Leis et al., 2014; Sun et al., 2014; Li et al., 2017). Therefore, it is not surprising that *Piromyces* was an important feature in predicting methane emissions based on our findings. We have shown further that there is a low and positive association between the abundance of *Piromyces* and methane emission in our data, in alignment with the existing knowledge.

*Succinivibrionaceae UCG-002* belongs to the bacterial family *Succinivibrionaceae*, which is known for the ability to produce succinate from substrates like hydrogen (Lee et al., 1999), which is also needed for methanogens to produce methane. In previous studies, an increased abundance of *Succinivibrionaceae UCG-002* has been associated with low methane emission in ruminants (Wei et al., 2022). Similar negative associations were also established between the family *Succinivibrionaceae* and methane production (Wallace et al., 2015). However, statistical tests that were used to identify such correlation are not able to handle the complex non-linear relationships as shown in our results. In contrast, our analysis showed that the relationship between *Succinivibrionaceae UCG-002* and methane emission is rather complex, instead of a simple negative association.

*Acetobacter*, an acetogen genus identified in the Merged dataset is characterized by its ability to produce acetate by oxidizing sugars (Balch et al., 1977). According to Lyons et al. (2018), a small amount of oxygen can be infused into the rumen fluid during feeding, drinking, or rumination. Such oxygen can be utilized by *Acetobacter*, which might lead to an anaerobic environment that promotes the growth of anaerobic archaea, such as most of methanogens. Previously, using statistical tests, Cunha et al. (2019) reported a positive association between the abundance of *Acetobacter* and methane production by heifers. However, similarly, though *Acetobacter* was also considered an important feature for methane prediction in our study, the direction of association was more complex according to our findings.

Our results indicated that certain herd locations were highly predictive features for methane emission. Animals from a few locations had notably high or low methane production conditions, which may be due to the fact that herd location encompasses many other variables, such as unmeasured dietary compositions, living environment, climate, husbandry regime, and genetic background of the host animals. Similar findings have been established in humans and other animals (Gomez et al., 2015; Van Treuren et al., 2015; Mobeen et al., 2018; Goertz et al., 2019). Unfortunately, with the available data in our study, it is impossible to determine the fundamental differences between herds.

When available, feed composition intake such as lipid intake, neutral-detergent fiber intake, and dry matter intake were also important for predicting methane emission. Dry matter intake (DMI) is the feed intake when the water content is excluded. The positive relationship between DMI and methane emission has been well-established for a century (Lakamp et al., 2022). This relationship can be succinctly explained: as ruminants consume higher quantities of dry matter, there are more substrates available for microbial fermentation, consequently leading to increased methane production. The mitigation effect of lipids for methane production is also known. Lipid supplementation has been reviewed as a potential strategy to reduce methane emissions from ruminants (Beauchemin et al., 2008; Hook et al., 2010; Knapp et al., 2014). As for neutral-detergent fiber (NDF), Hatew et al. (2016) previously reported a reduced *CH*_4_ emission with increased maturity of whole-plant maize, which has a decreased NDF content. Our results confirmed that these feed compositions are important for cow methane emission.

In the regression task, random forest regression (RF regression) exhibited superior performance during testing compared to other regression algorithms, especially linear regressors. The decreasing performance in the Difford and Wallace datasets from adding microbiome on top of biological metadata features suggests that adding microbial features does not always improve the prediction outcomes. This could be attributed to the increased dimension of search space and the complexity of the problem, which might overweight the added value from microbial features. However, RF regression was able to overcome this problem and learn from the microbial features, demonstrated by our results in the Merged dataset. These findings underscore the complex nature of the methane emission prediction problem.

Future research should decompose the compound variable “herd location” and pinpoint the actual differences between different herds from different farms or geographical locations. Moreover, evaluating the emission mitigation potential *Piromyces, Succinivibrionaceae UCG-002* and *Acetobacter* through modulation with dietary intervention is promising.

In conclusion, in this paper we identified three methane microbial biomarkers in the rumen microbiome: *Piromyces, Succinivibrionaceae UCG-002*, and *Acetobacter*. We also showed that herd location is a dominant feature for predicting methane emissions. Feed composition intake, such as DM intake, lipid intake and NDF intake were predictive as well. The superior performance of RF regression and later visualization indicated that the relationship between microbial OTU abundance and methane production could be non-linear. Overall, we showed that supervised machine learning can identify potential microbial markers for cow methane emission, similar to its use in human microbiome studies. Our findings of important microbial features can facilitate the design of microbiome-based precision feed to reduce methane emissions from ruminants and alleviate the climate crisis.

## Data availability statement

Publicly available datasets were analyzed in this study. This data can be found at NCBI, accession numbers: ERP110230 and PRJNA517480.

## Author contributions

CP: Conceptualization, Data curation, Formal analysis, Investigation, Methodology, Visualization, Writing – original draft, Writing – review & editing. AM: Conceptualization, Investigation, Project administration, Supervision, Writing – review & editing. TA: Conceptualization, Investigation, Project administration, Supervision, Writing – review & editing.
