# Supplementary figures and images for: Unveiling microbial biomarkers of ruminant methane emission through machine learning
Source: Front Microbiol. 2023 Dec 8;14:1308363. doi: 10.3389/fmicb.2023.1308363 (PMC10749206; doi:10.3389/fmicb.2023.1308363)

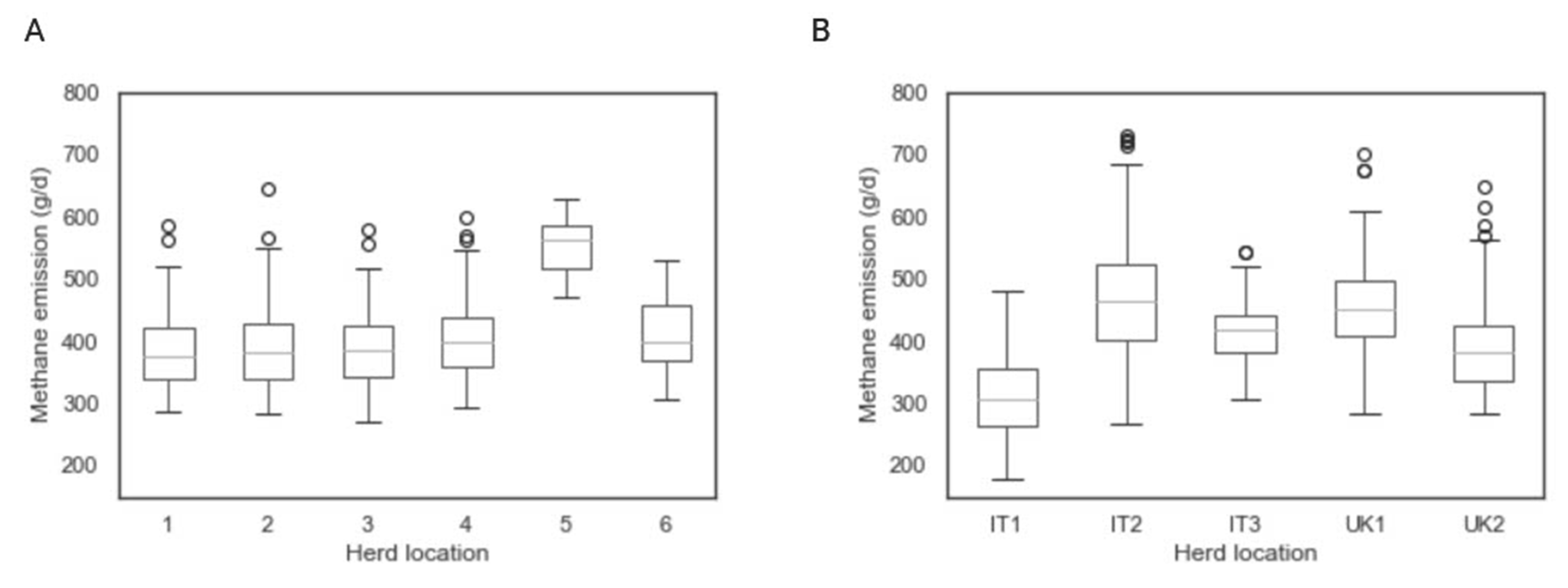

Supplement: Supplementary file 5 [file Image_2.JPEG]

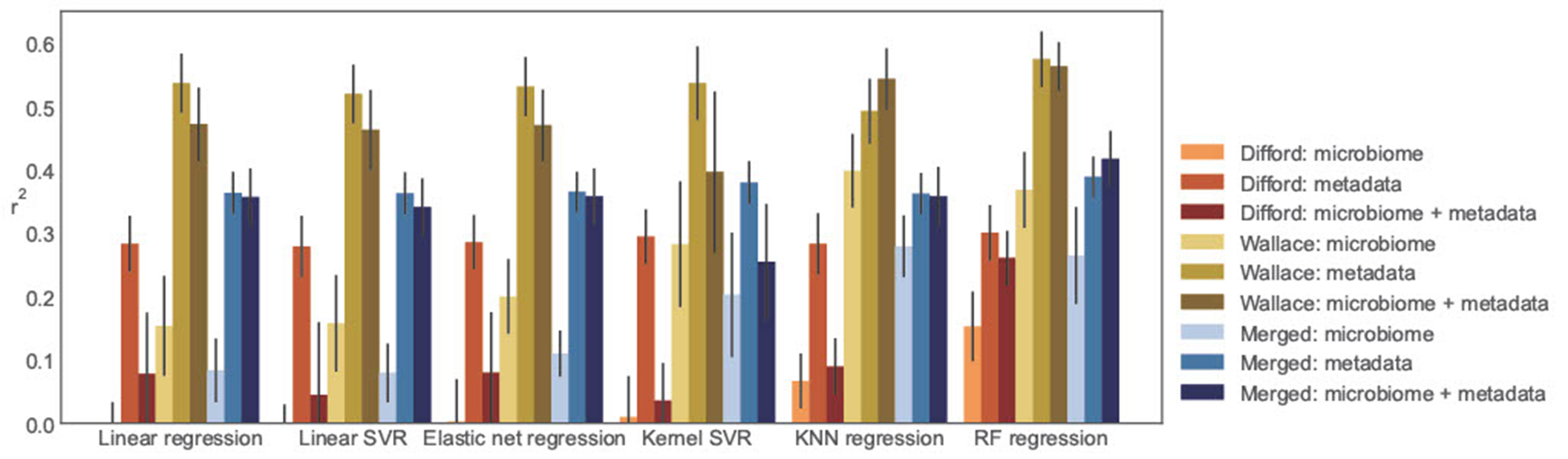

Supplement: Supplementary file 6 [file Image_3.JPEG]
